# Impact of a multi-component implementation strategy to increase outdoor free play opportunities in early childhood education and care (ECEC) services: the get outside get active (GOGA) randomised controlled trial

**DOI:** 10.1186/s12966-025-01749-0

**Published:** 2025-05-01

**Authors:** Sze Lin Yoong, Nicole Pearson, Luke Giles, Hannah Lamont, Luke Wolfenden, Jannah Jones, Christophe Lecathelinais, Patti-Jean Naylor, Anthony Okely, Nicole Nathan, Kathryn Reilly, Rebecca Lorch, John Wiggers, Jacklyn Jackson, Melanie Lum, Karen Gillham, Alice Grady

**Affiliations:** 1 Global Centre for Preventive Health and Nutrition, Institute for Health Transformation, School of Health and Social Development, Deakin University, 1 Gheringhap Street, Geelong, VIC 3220 Australia; 2 School of Medicine and Public Health, College of Health, Medicine and Wellbeing, University of Newcastle, University Drive, Callaghan, NSW 2308 Australia; 3 National Centre of Implementation Science, University of Newcastle, University Drive, Callaghan, NSW 2308 Australia; 4 Hunter Medical Research Institute, New Lambton Heights, NSW 2305 Australia; 5 Hunter New England Population Health, Wallsend, NSW 2287 Australia; 6 School of Exercise Science, Physical and Health Education, University of Victoria, STN CSC, Mackinnon 120, PO Box 1700, Victoria, BC V8W 2Y2 Canada; 7 Early Start, Faculty of the Arts, Social Sciences and Humanities, University of Wollongong, Wollongong, NSW 2522 Australia; 8 School of Health and Society, Faculty of the Arts, Social Sciences and Humanities, University of Wollongong, Wollongong, NSW 2522 Australia; 9 Western Norway University of Applied Sciences, Sogndal, Norway

**Keywords:** Outdoor play, Free play, Physical activity, Indoor-outdoor, Early childhood education and care, Implementation trial, Randomised controlled trial

## Abstract

**Background:**

Increased outdoor free play is associated with health and developmental benefits for preschool-aged children. It is therefore recommended that early childhood education and care (ECEC) services provide increased time for outdoor free play. This study seeks to understand the impact of a multi-component implementation strategy (Get Outside Get Active) on ECEC service provision of opportunities for outdoor free play.

**Methods:**

This was a parallel-group randomised controlled trial involving 84 ECEC services located in one region of New South Wales, Australia. Forty-one services were randomised to a 6-month multi-component implementation strategy or to a usual care group (*n* = 43). To increase total scheduled outdoor free play time, services were supported to modify their routines to increase provision of outdoor free play and/or indoor-outdoor free play opportunities (whereby children are allowed to move freely between indoor and outdoor spaces). The primary trial outcome, mean minutes per day of outdoor free play opportunities provided in ECEC services, was measured at baseline, 6-months (primary endpoint), and 18-months. Secondary outcomes were mean minutes of indoor-outdoor free play only and proportion implementing indoor-outdoor free play for the full day. The quality of the movement environment was assessed using direct observations in 30 ECECs at 6 months only.

**Results:**

At 6 months, the intervention group showed a significant increase in mean daily minutes of outdoor free play (61.3 min; 95% CI 2.5 to 120.01; *p* = 0.041) and indoor-outdoor free play (59.1 min; 95% CI 9.1 to 109.1; *p* = 0.021) relative to the control group. However, no significant between-group differences were observed at 18 months. The proportion implementing a full-day indoor-outdoor program (OR 1.97; 95% CI 0.81 to 4.78; *p* = 0.196) and the quality of movement environments did not differ between groups at 6 months.

**Conclusions:**

The implementation strategy significantly increased outdoor free play opportunities in ECEC services post-intervention, though the between group effects were not sustained at 18 months. Future research should focus on ensuring the long-term impact of implementation strategies and understanding the factors driving changes in control group behaviour.

**Trial registration:**

This trial was prospectively registered with the Australian New Zealand Clinical Trials Registry (ACTRN12621000987864).

**Supplementary Information:**

The online version contains supplementary material available at 10.1186/s12966-025-01749-0.

## Background

Outdoor play is recognised as important to support children’s physical, emotional, and cognitive development [1, 2], providing opportunities to develop gross and fine motor skills and promote creative play and autonomy [3]. Outdoor play time in children is associated with higher moderate-to vigorous-intensity physical activity (MVPA) and cardiorespiratory fitness [2], and benefits concentration, cognitive function, and memory [4].

The time available for outdoor play in young children (3–6 years) however has declined over the last 20 years in many developed countries [5, 6]. Leading international organisations [7, 8], recommend increasing opportunities for child self-directed (‘free’) play outdoors in all settings including home, schools, early childhood education and care (ECEC) services and the community [3].

ECEC services are an important setting to facilitate outdoor free play. In many countries, these services are where young children spend a significant portion of their day (10 h/day on average), providing a safe and developmentally appropriate environment to promote play [9]. Research suggests that increased outdoor free play can also improve the quality of the ECEC environment in ways that are supportive of children’s physical, development and movement skills by fostering more active, exploratory, and socially interactive behaviours between children and carers [10]. For example, an Australian cross-sectional study with 11 ECECs and 110 educators found that increased outdoor free play opportunities were associated with improvements in ECEC physical activity promoting environments, specifically around child-educator interactions and supporting reorienting of the outdoor environment to support child physical development [10]. Despite the potential benefits of outdoor play, children spend less than half of their time attending ECEC services in outdoor play [11, 12].

Accreditation standards and best-practice guidelines for the ECEC sector therefore recommend that services implement routines to increase outdoor free play [13, 14]. Although there is no guidance around the specific amount of outdoor free play provided in ECEC, current guidelines recommend that ‘more is better’. Evidence suggests that ECEC services can facilitate outdoor free play and increase child physical activity, by intentionally structuring routines to increase the time children are allowed to move freely between indoor and outdoor spaces (i.e. indoor-outdoor play) and/or increase total time available for outdoor free play [15, 16]. Although such recommendations align with general education and pedagogical approaches for the sector, ECECs report a number of barriers to planning such routines [17]. These include challenges with staffing ratios, orientating the service layout to facilitate transitions outside, child safety and risky play concerns, and challenges with size and orientation of the outdoor space [17, 18]. To maximise potential child benefits, there is a need to understand what strategies are effective to support implementation of routines that are conducive to increasing opportunities for outdoor free play in ECEC. A recent Cochrane systematic review exploring ECEC-based implementation strategies, however, identified an absence of trials specifically exploring this [19].

### Objectives

This study aimed to assess the impact of a multi-component implementation strategy (the Get Outside Get Active (GOGA) program) on opportunities for child outdoor free play in ECEC services, relative to usual care at 6- (post-strategy) and 18-months follow up. Given previous findings of an association between outdoor free play and improved physical activity environments, we also measured ECEC physical activity environment at 6-months via direct observation.

### Methods

The study was reported in accordance with the CONSORT standards for randomised controlled trials (RCTs) [20] and is consistent with best practice guidance for undertaking implementation trials [21]. A detailed protocol for this trial has been previously published [22]. Additional file 1 contains the CONSORT checklist. A TIDieR Checklist can be found in Additional file 2.

### Study design and setting

The study employed a two-arm parallel-group RCT. Eighty-nine ECEC services in the HNE region of New South Wales (NSW), Australia were recruited and randomised to receive either a 6-month multicomponent implementation strategy or usual care. The study assessed between-group differences in the mean minutes children were provided with opportunity for outdoor free play per week while attending ECEC services, assessed via a Free Play Record (FPR; educator self-reported measure). Data collection and implementation support were staggered over several months. Primary outcome data were collected at three time points: (1) baseline (between September 2021- April 2022); (2) following the delivery of the 6-month support (between May 2022– January 2023, primary timepoint); and (3) approximately 18-months post-baseline (November 2023– January 2024) to determine long-term impact after implementation support had ceased. For a subsample of services located within 50 km of the primary research site (Wallsend, NSW; *n* = 30), research assistants attended the services at 6-months to conduct direct observations of the quality of the ECEC physical activity environment.

### Context

This study was conducted while active measures were in place to reduce transmission of COVID-19. In this region, there was period of lockdowns of varying lengths for different areas from 5th August 2021 to 17th October 2021. In July 2021, recommendations from the NSW Department of Health for education settings (including ECEC services) were released which recommended services facilitate outdoor play and promote indoor-outdoor play to increase social distancing [23]. Our previous three-arm trial however suggests that dissemination of these recommendations to ECEC services via video and email was insufficient to increase implementation of outdoor-only and indoor-outdoor free play [24]. Thus, at the time of study initiation, we believe the recommendations in itself would not have been sufficient to influence the outcomes assessed in this study [24].

### Participants and recruitment

#### Sampling frame

A list of all centre-based ECEC services (long day care and preschools) located in the HNE Local Health District (HNELHD) of NSW, Australia (*N* = 444) was accessed via a central database provided by the NSW Ministry of Health [25] and served as the sampling frame. The HNELHD area encompasses major metropolitan centres and regional communities, with 14% located in remote communities [26].

### Eligibility

Eligible ECEC services were required to schedule at least one session of indoor-only free play across five consecutive days in a week. Indoor-only sessions were defined as periods of free play where children were unable to access the outdoor environment. Services were excluded if they were participating in any other trial related to improving physical activity practices, catered exclusively for children with special needs, were an occasional care, mobile, family day care, or a Department of Education operated service (representing less than 10% of services).

### Recruitment procedures

A pre-recruitment telephone call (*N* = 225) was undertaken by the research team to assess eligibility in February-May 2021. Following this, services were emailed information statements and consent forms outlining study requirements and inviting participation. Recruitment of services occurred in random order and was overseen by an experienced trial coordinator (KR). To maximise participation, researchers employed an evidence-based recruitment strategy which included multiple contacts with follow up by the same team member, and providing flexible completion of data collection tools [27].

### Randomisation and blinding

An independent statistician (CL) used a computerised random number function to block randomise services in a 1:1 ratio to either the intervention or control group. Block randomisation (2, 4 or 6) was used to ensure group allocation was approximately equal. Allocation was stratified by service type (long day care service or preschool) and service size (small < 80 child enrolments or large ≥ 80 child enrolments) given an association between these factors and the implementation of indoor-outdoor free play routines [17]. Randomisation was undertaken post baseline data collection and ECEC service managers were notified of group allocation following this.

The trial was conducted as an open trial where ECEC services and those delivering the intervention were aware of the allocation. Outcome assessors and the statistician were blinded.

### Evidence-based practice (EBP)

The targeted practice was for ECEC services to provide children with increased opportunities for outdoor free play. The benefits of outdoor free play are well described in the literature [1, 2] and the accreditation body for ECECs in Australia also promotes the benefits of outdoor play for both physical activity and promoting quality learning experiences [28]. For services, this included changing routines to incorporate additional outdoor-only and/or indoor-outdoor free play. This is supported by two RCTs reporting that restructuring ECEC routines to increase the frequency and duration of access to outdoor play areas can have positive effects on child MVPA [15, 16]. The restructuring of ECEC routines in this way aligns with accreditation standards for the sector and recommendations within state health promotion programs [29, 30].

### Implementation strategy

The GOGA program was a 6-month multi-component implementation strategy designed to increase ECEC service provision of outdoor free play opportunities. ECEC services were supported to identify free play sessions in their routines designated for indoor-only play and converting them to indoor-outdoor or outdoor-only free play sessions. Services were asked not to alter any play opportunities where adult-facilitated activities were undertaken. No active support was provided post 6-months. The implementation strategies were delivered by experienced health promotion officers (HPO) employed by HNELHD. Strategies were typically delivered virtually (i.e. via online meetings).

### Theoretical framework

The development of the GOGA intervention was informed by the Behaviour Change Wheel [31] and the Theoretical Domain Frameworks (TDF) [32] and overseen by an expert advisory group consisting of implementation and behavioural scientists, physical activity specialists, local health promotion team, representatives from early childhood organisations and health service managers. The TDF summarises 33 theories and 128 constructs which can influence implementation behaviour. The application of this framework has been described in the study protocol [22]. We assessed barriers to implementation of outdoor free play opportunities via: (a) a systematic review [33]; (b) a quantitative survey with ECEC services [17]; and (c) consultation with service managers and educators from five ECEC services. Following identification of barriers, we undertook a process of mapping the barriers against the capability, opportunity, and motivation model of behaviour (COM-B) of the Behaviour Change Wheel to select intervention functions [34]. Finally, the Theory and Techniques tool [35] was used to select appropriate Behaviour Change Techniques (see Table 1). The Expert Recommendations for Implementing Change taxonomy [36] was used to describe implementation strategies.

**Table 1:** Overview of mapping process to identify implementation strategies and behaviour change techniques (BCTs) to address identified barriers

Target behaviour: Amending all indoor-only free play time to either indoor-outdoor or outdoor-only to increase duration children allowed to access outdoor areas				
**Barriers reported by ECEC services**	**COM-B and (TDF)**	**Intervention function**	**BCTs applied**	**Implementation strategy (modality)**
Staff lack of awareness of the benefits of outdoor play	Capability– psychological (Knowledge)	Environmental restructuring Education	Information about health consequences Information about social and environmental consequences Instruction on how to perform the behaviour	Develop and distribute educational materials (email and hard copies)
Lack of time to implement Perceived priority for the service	Motivation- reflective (Intentions, Beliefs about capabilities)	Enablement Persuasion	Verbal persuasion about capability Focus on past success Discrepancy between current behaviour and goal Social support	Conduct educational outreach visits (online meetings, phone calls) Identify and prepare GOGA champions (email and online meeting)
Lack of awareness of alignment with COVID-19 Guidelines for NSW ECEC services and the National Quality Framework	Motivation- reflective (Social /professional role and identify)	Enablement Education	Credible source Information about social and environmental consequences	Conduct educational sessions (online meeting) Develop and distribute educational materials (email and hard copies) Provide local centralized technical assistance
Logistical arrangements: Including service staffing, service layout not conducive to outdoor play Lack of specific plans to put the intervention into effect	Capability–physical opportunity (Environmental context and resources)	Environmental restructuring Enablement	Restructuring the physical environment Goal setting (behaviour) Problem solving Action planning	Conduct educational sessions (online meeting) Provide local centralized technical assistance (online meetings and phone calls) Performance review and feedback (online meeting, phone) Develop a formal implementation blueprint (online meetings)
Perceived lack of support from service managers/peers/ parents	Opportunity-social (Social influences)	Persuasion	Social support (practical) Information about others’ approval	Identify and prepare GOGA champions (email and online meeting) Develop and distribute educational materials (email and hard copies)
Unable to identify and attribute positive changes/outcomes to implementation efforts	Motivation- reflection (Intentions, Beliefs about consequences)	Persuasion Enablement	Review behaviour goals Self-monitoring of behaviour Feedback on outcome of behaviour	Performance review and feedback (feedback summary (email), online meeting, phone and face-to-face (*n* = 1))

ECEC: Early childhood education and care; TDF: Theoretical Domains Framework; COM-B: Capability, opportunity, and motivation model of behaviour; GOGA: Get Outside Get Active

### Identify and prepare GOGA champion

ECEC services were asked to nominate a GOGA champion to support implementation. A position description was provided to ECEC services. Specific expectations included leading the delivery of an action plan, monitoring implementation, and participating in audit and feedback activities.

### Develop and distribute educational materials

The service manager and GOGA champion were provided with hardcopy and electronic information packs. These contained educational materials regarding how the evidence-based practice aligned with ECEC accreditation standards and social distancing recommendations released by the NSW Department of Education. ECEC services received case studies, fact sheets addressing common barriers, family newsletter snippets, footprint floor stickers (acting as a prompt for staff and children to get outside) and four newsletters throughout the 6-month intervention.

### Conduct educational sessions

Two one-hour online meetings were undertaken with the service manager and GOGA champion and facilitated by the HPO. The first meeting was to introduce the implementation intervention, provide information to reinforce the importance of increased outdoor free play opportunities, identify alignment with service philosophy and priorities, and to support action planning. The service manager and GOGA champion were encouraged to organise a second educational session, co-facilitated by the service manager and HPO, with all service educators. As an alternative, a pre-recorded educational video could be played at an ECEC staff meeting.

### Develop a formal implementation blueprint (or action plan)

During the first meeting, the HPO, service manager, and GOGA champion developed an electronic action plan, setting clear goals and strategies to increase outdoor free play opportunities. The action plan was part of a workbook that included: space to identify and document motivations and anticipated benefits of increasing outdoor free play opportunities, document suitable long- and short-term goals, an environmental checklist, problem solving tips, and sample actions. The service manager and GOGA champion were encouraged to communicate and develop the action plan in consultation with all educators and monitor ongoing progress towards goals. A meeting to finalise the action plan was undertaken after the two educational meetings.

### Provide local centralized technical assistance

The HPOs provided up to two 25–40-minute support meetings for the GOGA champion to review progress with goals and actions, and to problem solve and set any new goals or actions, if required. Support was tailored according to the preferences, needs and/or barriers of each service. Additional support meetings were scheduled if needed.

### Performance review and feedback

ECEC services received written and verbal feedback on their performance against baseline data mid-intervention (3-months). The GOGA champion completed an observation tool at their service to observe and record the amount of indoor, outdoor, and indoor-outdoor free play opportunities provided over a usual 5-day period. Based on this data, a feedback report was provided which compared their current practice to baseline. A phone call or video conference was undertaken by the HPO to discuss feedback and provide recognition for achievements and/ or facilitate problem solving and progression of goals.

### Control group

ECEC services allocated to the control group received ‘usual’ implementation support delivered as part of statewide obesity prevention programs. This included the passive provision of information and resources via a website and email contact, including factsheets, example policies and templates, related to physical activity in general, but not outdoor free play specifically.

### Data collection and measures

#### Primary outcome: mean minutes per day ECEC services provide children with the opportunity for outdoor free play in a usual week

The primary trial outcome was fidelity of implementation, which was operationalised as the mean minutes ECEC services provided children with outdoor free play opportunities per day in care at 6-months follow up. This was calculated by adding the minutes of outdoor-only free play and of indoor-outdoor free play opportunities per day. These data were collected using a free play record (FPR), which was a self-reported log, at three time points: baseline, 6-months and 18-months follow up [37, 38]. A selected educator (usually the GOGA champion) from the classroom reported the start and end time of each free play session, as well as whether the session were indoor-only, outdoor-only, or indoor-outdoor, for five consecutive days to provide a sample of a typical week. The research team followed up where additional information was needed to ensure data was captured for five days. The FPR was adapted from an existing measure of outdoor play in ECEC services and previously used by the team in primary schools [39]. Our unpublished data indicates that the self-reported FPR overestimates opportunities for outdoor free play (the primary outcome) by 7.6 min per day (95% CI -15, 15) when compared to observations (unpublished data). The FPR also asked educators to report on whether it was sunny, overcast, raining, storming, strong winds, or extreme heat for each day.

### Secondary outcomes

#### Mean minutes ECEC services provide children with opportunities for indoor-outdoor free play per day in a usual week

The FPR was also used to calculate mean minutes of indoor-outdoor free play per day for a usual week. This was a post-hoc change to the registered outcomes which proposed calculating this as a percentage of free play time. This was undertaken to better understand whether change in outdoor opportunities were directly attributed to increases in scheduling of indoor-outdoor free play.

#### Proportion of ECEC services with indoor-outdoor and outdoor-only routines for an entire week

This was a dichotomous outcome. Services were classified as ‘implementing’ if children were provided with the opportunity to access the outdoor environment during every session of free play (i.e. no indoor-only free play during service hours). If a service reported offering any indoor-only free play during service hours, they were classified as ‘not implementing’. The number implementing was divided by total number in the group and proportion compared between groups.

#### Quality of the ECEC physical activity environment

For 30 ECEC services, two trained research staff observed the service environment for one day at 6-months follow-up using Movement Environment Rating Scale (MOVERS). MOVERS is a validated, observational tool that examines ECEC physical activity environments related to children’s physical development and movement [40]. MOVERS focuses on the influence of the educators and physical activity learning experiences offered to the children, which have been previously reported to be associated with increased outdoor free play [10]. MOVERS includes 11 items across 4 subscales; (i) Curriculum, environment and resources for physical development (4 items); (ii) Pedagogy for physical development (3 items); (iii) Supporting physical activity and critical thinking (3 items); and (iv) Parents/carers and staff interaction (1 item) [40]. Each item is scored on a 7-point scale (1 = inadequate, 3 = minimal, 5 = good, 7 = excellent). Higher scores on the MOVERS scale [40] is positively associated with MVPA in pre-schoolers (β = 0.08 (95% CI: 0.01, 0.14); β = 0.07 (95% CI: 0.03, 0.12)) [41]. MOVERS has also been shown to have good test-retest reliability (weighted Kappa = 0.91; percentage agreement = 69–100%), internal consistency (Cronback’s α = 0.94),concurrent validity (Spearman’s ρ = 0.57–0.87) when compared to other established measures in ECEC (i.e. the Environment and Policy Assessment and Observation) [42]. It is also associated with increased light and moderate physical activity levels [43].

### Other data collection

#### ECEC characteristics

Data relating to service type, enrolment numbersa, hours of operation, and number of educators employed were collected via a baseline survey of ECEC service managers and educators using items sourced from previous surveys of ECEC services [44]. Service postcode was used to assess socioeconomic status (SES) of the area and rurality. Where this was not an appropriate indication of SES (i.e. university or military base), the closest suburb postcode was used instead.

#### Fidelity of intervention delivery

Fidelity of delivering the GOGA implementation strategies were measured using monitoring instruments developed by the research and health promotion team. Details of adaptations and modifications made to the implementation strategy have been published elsewhere [45].

#### Adverse effects

As an increase in child outdoor play could potentially increase the risk of child injury [46], service managers in both groups were asked to report on the number of child injuries (including moderate or severe injuries) that had occurred in the past 6 months.

#### Co-intervention

Service managers were asked if they received any other education and/or support to implement physical activity programs in the last 6 months and to report on the type of support received.

#### Analysis and sample size

Analyses were performed using SAS (version 9.3) statistical software, following an intention-to-treat approach, with ECEC services as the unit of analysis. Descriptive statistics were calculated for ECEC service characteristics at baseline. Intervention effects on the primary and secondary trial outcomes at 6- and 18-months were assessed using a linear mixed effects regression model, which included fixed effects for the treatment group (intervention vs. control), the baseline value of the outcome and variables that are prognostic of the outcome (service size and geographical region). Multiple imputations were performed for those not providing baseline or follow up data in accordance with the recommendation by White and colleagues [47] and presented as the primary analysis. Multiple imputation involves imputing missing data with plausible values based on already collected data. For this study, this was done 20 times, and each imputed dataset was analysed and combined using Rubin’s rules.

To assess potential impact of the intervention on ECEC service environments supporting children’s physical development and movement, we undertook a post intervention assessment using MOVERS. The score for each of the 11 items within MOVERS were summed to create an overall score (a possible total score of 77) and scores within each subscale were also calculated. The overall and subscale scores were compared between groups using a t-test analyses.

#### Sample size calculation

We intended to recruit 100 ECEC services. This would enable us to detect an absolute difference of 25.34 min/day in the time children have the opportunity to spend in outdoor environments during free play (primary outcome) assuming a standard deviation of 43 min with 80% power and an alpha of 0.05 based on previous research [48]. We powered to detect a difference of approximately 25 min of outdoor free play time per day as this would increase total outdoor free play time from on average 40 min to > 1 h for services. Studies suggest that an increase of one hour of outdoor free play time can result in an additional 10 min of MVPA [11] which has clinically significant, beneficial effects on fat mass and peak bone mass [49, 50].

## Results

**Fig. 1:**
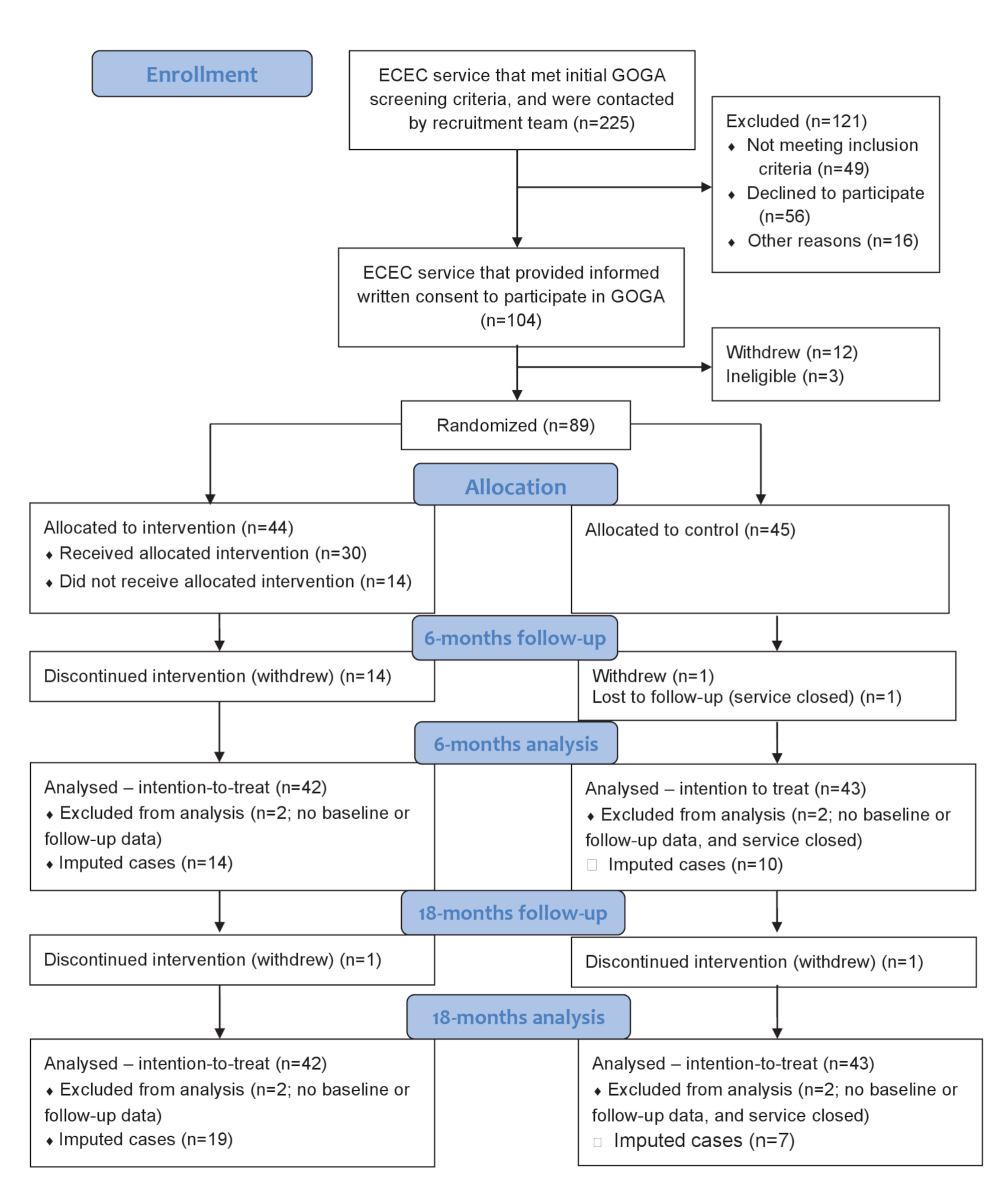
CONSORT flow diagram

Overall, 176 ECEC services were identified as eligible to participate, and 104 services consented (59%) to participate. Prior to randomisation, twelve services withdrew and three more were deemed ineligible. A total of 89 services were randomised to receive either the intervention (*n* = 44) or control (*n* = 45) (see Fig. 1 for CONSORT flow diagram). Five services did not provide any baseline data.

At baseline, over 70% of services were long day care services, with the majority in the most disadvantaged areas within the state (see Table 2). No significant differences were found in service characteristics between consenters and non-consenters.

**Table 2:** Baseline characteristics of ECEC services (*n* = 89)

	Intervention (*n* = 44)	Control (*n* = 45)
**Service Type,** ***N*** **(%)**		
Preschool	13 (29.6)	12 (26.7)
Long Day Care	31 (70.5)	33 (73.3)
**Service sociodemographic position**, N (%)*		
Least disadvantaged	12 (29.9)	14 (31.1)
Most disadvantaged	29 (70.0)	29 (67.4)
**Service Location**, N (%)**		
Major city	21 (51.2)	22 (51.1)
Inner/Outer Regional or remote Australia	20 (48.8)	21 (49.9)
**Total number of children in care per day**,** Mean (SD)**	53.6 (29.2)	47.8 (22.8)
**Number of children aged 3–6 years in care per day**,** Mean (SD)**	37.2 (17.9)	31.4 (13.4)
**Open 5 days per week**,** N (%)**	39 (88.6)	42 (93.3)
**Service open hours per day**,** Mean (SD)**	10.04 (1.88)	10.11 (1.76)
**Number of full-time educators at the service**,** Mean (SD)**	8.95 (7.73)	7.00 (5.12)
**Number of part-time educators at the service**,** Mean (SD)**	6.75 (6.88)	5.89 (5.12)
**Number of casual educators at the service**,** Mean (SD)**	2.55 (2.13)	2.11 (2.17)
**Years centre manager has been in current role**,** Mean (SD)**	8.08 (8.34)	7.05 (7.59)
**Centre manager highest relevant qualification**,** N (%)**		
Post graduate degree or Bachelor degree	31 (75.6)	23 (53.5)
Advanced diploma, diploma or graduate certificate	10 (24.4)	20 (46.5)

SD: Standard deviation; * This was assessed using service postcode and categorised as using the Socio-economic Indexes for Area into top 50% and lower 50%. ** This was assessed using service postcode and categorised using the Accessibility/Remoteness Index of Australia

At 6-months follow up, 60 services (71%) provided data on the primary outcome and secondary outcomes (27 intervention and 33 control). All but one service provided the data required (i.e. five days). Additionally, a subsample of 30 services (*n* = 12 intervention, *n* = 18 control) consented (88% of services in the targeted area) to a one-day observation at 6-months follow up to assess ECEC environments supporting children’s physical development and movement. At 18-months, 58 (69%) ECEC services returned their FPR (22 intervention and 36 control).

Of the control services, 50% (241/490) of the days were reported as sunny, while in the intervention services, 57% (232/404) of the days were reported as sunny at 6-months. This was similar at baseline for control (49%) and intervention groups (57%). We did not collect this data at 18-months.

### Primary outcome

#### Mean minutes that ECEC services provide children with the opportunity for outdoor free play per day across a usual week

At 6-months, there was a significant increase in mean minutes of outdoor free play opportunities per day in the intervention group relative to usual care (mean difference: 70.95 min; 95% CI 11.9 to 130.0; *p* = 0.019), after controlling for baseline, service SES and geographical location (see Table 3). At 18-months, this was no longer significant, and the effect was favouring the control group (mean difference − 21.9 min; 95% CI -76.6, 32.9; *p* = 0.43).

**Table 3:** Baseline, 6-months and 18-months opportunities for outdoor and indoor-outdoor free play

Measure	Intervention (*n* = 42)			Control (*n* = 43)			Multiple imputation analysis^a^ Baseline versus 6-months		Multiple imputation analysis^a^ Baseline versus 18-months	
	Baseline mean (SD)	6-months mean (SD)	18-months mean (SD)	Baseline mean (SD)	6-months mean (SD)	18-months mean (SD)	Relative effect size		Relative effect size	
Mean minutes							Mean difference (95% CI)	*P* value	Mean difference (95% CI)	*P* value
Outdoor free play opportunities (average minutes per day across five days)	227.8 (104.1)	299.3 (134.2)	280.6 (107.6)	206.6 (114.3)	232.6 (106.3)	295.0 (127.6)	61.3 (2.5; 120.1)	0.041	-21.9 (-76.6; 32.9)	0.43
Indoor-outdoor free play (average minutes per day across five days)	78.3 (99.3)	136.8 (127.8)	170.3 (141.2)	75.1 (92.1)	99.0 (104.4)	130.5 (166.2)	59.1 (9.1; 109.1)	0.021	36.5 (-31.5; 104.5)	0.29

^a^Adjusted multiple imputation analysis controlling for baseline data, service socioeconomic status and location

### Secondary outcomes

#### Mean minutes that ECEC services provide children with indoor-outdoor free play per day in a usual week

At 6-months, there was a statistically significant increase in average minutes ECECs provided indoor-outdoor free play in the intervention group relative to usual care (mean difference 66.4 min; 95% CI 11.3 to 121.5; *p* = 0.019), after controlling for baseline, service SES and geographical location (see Table 3). This was no longer significant at 18-months (mean difference 36.5 min; 95% CI -31.5 to 104.5; *p* = 0.29).

#### Proportion of ECEC services with indoor-outdoor and outdoor-only routines for an entire week

There was no difference between groups in the proportion of services that were fully implementing at 6-months (51.2%, control 32% (OR 1.97; 95% CI 0.81 to 4.78; *p* = 0.196)) and 18-months (intervention 22.7%, control 19.4% (OR 1.22; 95% CI 0.33 to 4.45; *p* = 0.76)), after controlling for baseline service SES and geographical location.

#### Quality of the ECEC physical activity environment at 6-months

In the 30 services where observations were undertaken (*n* = 12 intervention and *n* = 18 control), there were no significant differences between groups for total scores (mean difference: 0.34; 95% CI -0.18, 0.86; *p* = 0.19) nor for any of the four subscales of the MOVERS tool: (1) curriculum, environment and resources for physical development (mean difference: 0.31; 95% CI -0.81, 0.64; *p* = 0.31); (2) pedagogy for physical development (0.06; 95% CI -0.5, 0.60; *p* = 0.82); (3) supporting physical activity and critical thinking (0.48; 95% CI -0.16, 1.13; *p* = 0.13); and (4) parent carer/staff interaction (0.83; 95% CI -0.09, 1.76; *p* = 0.076).

### Delivery of implementation strategies

Of the 41 intervention services, 100% received hardcopy and electronic information packs and had a dedicated HPO available to provide support. Over 80% received one educational visit and 86% had a dedicated GOGA champion (see Table 4). Less than 60% were provided with performance review and feedback and received the first support call.

**Table 4:** Number of services receiving each implementation strategy

Implementation strategies	Number receiving each strategy (*n* (%))
Develop and distribute educational materials	42 (100%)
Conduct educational outreach visits:	
Visit 1 Visit 2	34 (81.0%) 23 (54.8%)
Identify and prepare GOGA champions	36 (85.7%)
Develop a formal implementation blueprint	37 (88.1%)
Performance review and feedback	25 (59.5%)
Provide localised technical support (dedicated HPO support)	42 (100%)
Support call 1 Support call 2	27 (64.9%) 28 (66.7%)

### Adverse effects

There were no significant differences in the number of moderate (OR 0.88; 95% CI 0.30 to 2.56; *p* = 0.81) or severe (OR 1.29; 95% CI 0.28 to 5.94; *p* = 0.74) injuries reported by service managers at 6-months follow-up.

### Receipt of other physical activity intervention (co-intervention)

43% (*n* = 12) of control services reported receiving “any education and/or support to implement physical activity policies and practices in the last 6 months” at 6-months follow up. Approximately 17% reported receiving support with development of their physical activity policy, and to implement fundamental movement skills for children as part of the Munch & Move program (i.e. a state-wide funded program supporting physical activity and healthy eating in the ECEC setting).

## Discussion

This study found that a 6-month multi-component implementation strategy significantly improved mean minutes of outdoor free play opportunities provided by ECEC services to children in care by 61 min per day at 6-months. Although data collection was conducted in different seasons at baseline and follow up, the percentage of sunny days were similar at both time points, suggesting that this likely did not influence the observed outcomes. Our study also found no difference in reporting of child injuries between-groups, addressing reported concerns that increased outdoor play would increase risk of injury [51]. Such significant improvements post-intervention and the lack of adverse effect observed is consistent with that reported in a Cochrane review which found that a range of implementation strategies can improve delivery of physical activity interventions in ECEC services [19].

We did not find a statistically significant difference in MOVERS scores between the groups at 6-months even with the substantial increases in outdoor free play opportunities provided in intervention services. There were potentially promising effects on the components supporting physical activity and critical thinking, and parent/carer staff interaction although this study was not powered to detect a difference in those outcomes. Future prospective research assessing outdoor free play in ECEC should consider measuring such components specifically to provide a better understanding of how the broader ECEC physical activity environment may also be influenced.

The significant between-group effects of the program however were no longer present at 18-months follow up although intervention effects were largely preserved (6-months: mean minutes: 299.3, and 18-months: mean 280.6 min). The two trials measuring the impact of implementation strategies on PA outcomes in ECEC beyond 12 months have reported mixed effects [52, 53]. Both these studies however collected data immediately post-implementation strategy, whereas our study provides an indication of what happens once active support has been withdrawn. Anecdotal data from health promotion officers that delivered the intervention suggests that among intervention ECEC services there was some fatigue from engaging with some elements of the intervention, which may have accounted for higher dropouts at 18-months. Based on delivery data, it is possible that some of the intervention components that were challenging to deliver (including education sessions and performance feedback) could have been reduced to address this fatigue.

There could be several reasons for the lack of ongoing effect of the intervention. Firstly, the strategy drew attention to sector recommendations at the time to increase outdoor free play to minimise spread of COVID-19 [23]. As such, there may have been high motivation to implement such practices, which could account for the rapid increase and large effect size initially in the intervention group. The health promotion officers that delivered strategies were also particularly useful in supporting services to overcome any barriers to implementation during this period. There were large increases in the control group’s provision of outdoor free play opportunities in the period following 6-month data collection, where active COVID-19 precautions had largely been withdrawn. Over 40% of the control group in our current study also reported receiving some support to implement physical activity policies and practices at 6-months follow up which could have contributed to some of the increases observed in the period following this.

It is possible that asking services to self-report on provision of outdoor free play opportunities during this time may have prompted ECEC services in the control group to modify their practices to increase outdoor free play opportunities over time. The literature on self-monitoring with teachers suggests that it could be useful to enhance the delivery of positive education strategies although this was most effective when paired with other strategies including goal setting and delivery of prompts [54].A previous trial in ECEC also reported large improvements in the control group’s implementation of physical activity and nutrition practices in ECECs potentially due to effects of self-assessment via a telephone survey. [53].Future studies assessing the impact of data collection activities where educators are required to report activity on multiple occasions on targeted outcomes may be warranted to understand how this influences control group behaviour.

There were also some challenges with delivery of certain implementation strategies including the performance monitoring and feedback strategy. Although the audit tool was designed to be brief and easy to complete, this was often not fully completed which affected the ability to provide performance feedback and support ongoing monitoring which may be particularly important for sustainment. Our detailed monitoring of implementation strategy delivery at the BCT level found that 50% of services received the full dose of BCTs at all planned occasions [45], and multiple attempts were made to ensure that BCTs could be delivered at different occasions, where possible, which could explain the large effect size initially. Although the effects were broadly maintained within the intervention group at 6-months and 18 -months, it is possible that ongoing additional support could have resulted in larger effects within intervention ECEC services at 18-months.

The large effect sizes from the GOGA program observed at 6-months reinforce the importance of systematically assessing implementation barriers and designing strategies that target barriers to implementation on improving implementation outcomes. Collectively, the literature suggests [19] that implementation interventions including preparing champions, providing educational materials, training, developing formal implementation blueprints, action planning and performance review and feedback can improve implementation in the short term however the impact of individual strategies on its own may warrant further exploration.

Research by the team also identified other key factors influencing the sustainment of physical activity programs in ECEC including those linked to the processes domains (e.g. lack of professional development, lack of monitoring) and inner context (including high staff turnover, room and service layout) that need to be better addressed prior to withdrawing implementation support [55]. Given the small decline of effects in the intervention arm (~ 20 min), future studies should also focus on incorporating strategies that can target barriers to sustainment including embedding reengagement processes as part of usual procedures (i.e. annual policy reviews and communication with parents) and ensuring ease of self-assessment processes.

### Strengths and limitations

Strengths of the study include the randomised design, prospective registration, delivery of the intervention to many services and a longer term follow up. However, several study limitations are worth noting. First, the study relied on the self-report completion of a FPR by service managers or champions, which may have introduced biases such as social desirability bias. While the tool was adapted from a similar measure used in schools [39], it has not been validated in Australian ECECs. Further, it is likely that increased participant burden with the multiple data collection points could have impacted on accuracy of data collection at the later time points. Although we had originally intended to undertake direct observations, this was not possible due to COVID-19 restrictions. Future studies using direct observations of free play opportunities is needed to confirm our study findings. Second, there were challenges with delivery of the planned implementation strategies per protocol due to competing priorities. Fourteen ECECs withdrew from receiving the strategies throughout the 6 months due to competing priorities and high staff turnover, however still provided follow-up data. Although this reflects real world implementation challenges, it is possible the impact of the implementation strategy was underestimated. Thirdly, the study reported on ECEC provision of outdoor free play opportunities, and it is unknown the extent that children accessed the outdoor areas in our study. Future research could consider assessing the number of children accessing outdoor areas during these free play opportunities to better understand the impact on child behaviour. At follow up, there was a higher percentage of missing data for the intervention arm compared to the control arm at both time points. As multiple imputation relies on the assumption that the data is missing at random, it is possible there may be bias with imputing data based on those that have provided data at follow up. However, given the large effect size observed at 6-months (primary time point), we do not expect that this is likely to affect the overall conclusions. Lastly, the ECECs who consented to participating in our study reported providing relatively high amounts of outdoor free play at baseline suggesting that this sample may consist of those already interested in providing outdoor free play. It is likely that delivering such strategies in ECECs where baseline provision of outdoor free play was low could provide a better indication of the possible benefits of such strategies. The literature suggests that these are likely to be services who have lower resourcing in terms of full-time staff and limited play areas [56].

## Conclusions

The GOGA program significantly increased the provision of outdoor free play opportunities in ECEC services by > 60 min at 6-months follow-up, however this was not significant at 18-months largely due to improvements observed in the control group.

## Electronic supplementary material

Below is the link to the electronic supplementary material.

Supplementary Material 1

Supplementary Material 2

Supplementary Material 3

## Data Availability

The datasets used and/or analysed during the current study are available from the corresponding author on reasonable request.

## Abbreviations

BCT: Behaviour Change Technique
COM-B: Capacity, Opportunity & Motivation Models of Behaviour
ECEC: Early Childhood Education and Care
FPR: Free Play record
GOGA: Get Outside Get Active
HNE: Hunter New England
HNELHD: Hunter New England Local Health District
HPO: Health Promotion Officer
HREC: Human Research Ethics Committee
MOVERS: Movement Environment Rating Scale
MVPA: Moderate-to-Vigorous Physical Activity
NSW: New South Wales
RCT: Randomised Control Trial
SES: Socioeconomic Status
TDF: Theoretical Domains Framework
