# Supplementary material for: Impact of a multi-component implementation strategy to increase outdoor free play opportunities in early childhood education and care (ECEC) services: the get outside get active (GOGA) randomised controlled trial
Source: Int J Behav Nutr Phys Act. 2025 May 1;22:51. doi: 10.1186/s12966-025-01749-0 (PMC12046965; doi:10.1186/s12966-025-01749-0)
Supplement: Supplementary file 3 — Supplementary Material 3 [file 12966_2025_1749_MOESM3_ESM.docx]

**Additional file regarding sample**

*How the sample was recruited:*

The sampling frame consisted of 444 centre-based ECEC services located in one region in NSW Australia (Hunter New England Local Health District). Of these a subsample of 225 services were selected and a pre-recruitment telephone call (N=225) was undertaken by the research team to assess eligibility. Of those, 176 were eligible and all were invited to participate in the study via email and with multiple follow up calls. 89 of the 176 were randomized to either the intervention or control group.

*Representativeness of the target group:*

There were no differences in service demographics (SES, service size and rurality) between consenters and non-consenter indicating limited selection bias. There was an overrepresentation of those located in low SES regions compared to all services located in the HNE region (>70%).

*Missing data and how the analysed sample differed from the recruited sample:*

An intention-to-treat approach was undertaken with the analysis, where multiple imputation techniques were used to account for missing data and service withdrawals. This analysis was reported as the primary analysis.
